# Landmark-Based Wing Morphometrics for Three *Holotrichia* Beetle Species (Coleoptera, Scarabaeoidea)

**DOI:** 10.3390/biology14030317

**Published:** 2025-03-20

**Authors:** Pengliang Pan, Shibao Guo, Fangmei Zhang, Zhou Zhou

**Affiliations:** College of Agronomy, Xinyang Agriculture and Forestry University, Xinyang 464000, China; panzai-7@163.com (P.P.); 2008180026@xyafu.edu.cn (S.G.); 2015180005@xyafu.edu.cn (F.Z.)

**Keywords:** hind wings, *Holotrichia*, interspecific, intraspecific, morphology, scarab, wing junction, wing venation

## Abstract

The scarab beetle is an herbivorous pest that causes considerable damage to various agricultural crops. The characteristics of the hind wings have rarely been utilized as indicators for species identification, particularly within the genus *Holotrichia*. To evaluate the potential of hind wing traits in species identification and gender differentiation, we extracted 25 landmarks from 125 samples of three beetle species (*Holotrichia diomphalia*, *H. titanis*, and *H. oblita*) using TPSDig2 v2.31, with each hind wing image analyzed three times. These landmarks were employed to analyze variations in wing size and shape among species and sexes, and a cross-validation test was conducted in MorphoJ v1.06 to assess classification accuracy. The results demonstrate that both female and male samples exhibit significant differences in wing size and shape variations across species, but not between sexes. Allometry accounts for 16.92% and 25.35% of total shape variation in females and males, separately. After correcting for allometric effects, classification accuracy improves for these beetles. From further analysis, it can be observed that female wings exhibit a wider and shorter morphology in comparison to the more slender and elongated wings of males. In terms of interspecific differences, *H. oblita* females displayed narrow and elongated wings, whereas *H. diomphalia* females had a more rectangular wing shape. Among males, the degree of wing narrowness decreased in the order of *H. oblita*, *H. titanis*, and *H. diomphalia*.

## Simple Summary

Many species of scarabs are remarkably similar in appearance, with some species showing minimal sexual dimorphism, thereby complicating the differentiation between males and females. Geometric morphology techniques, which rely on landmark-based analysis, have proven effective in distinguishing not only closely related insect species but also different sexes within the same species. Traditionally, the shape and size of the hind wings of Coleoptera have not been considered a primary feature for taxonomic identification. However, the potential taxonomic significance of wing venation characteristics among scarabs within the same genus warrants further investigation. This study employs shape analysis techniques based on landmarks to conduct the spatial analysis of wing venation characteristics across three scarab species. The results indicate that both wing shape and size hold significant reference value for discriminating between species and sexes. After accounting for wing size, the accuracy of distinguishing female and male specimens exceeded 94.12% and 76.67%, respectively. The female wings exhibited a wider and shorter morphology compared to the more slender and elongated wings of males. In terms of interspecific differences, *H. oblita* females displayed narrow and elongated wings, whereas *H. diomphalia* females had a more rectangular wing shape. Among males, the degree of wing narrowness decreased in the order of *H. oblita*, *H. titanis*, and *H. diomphalia*. These findings underscore the substantial research value of geometric morphometrics in differentiating closely related insect species and their respective sexes.

## 1. Introduction

Scarab beetles (Coleoptera) represent economically significant pests that often inflict damage on a broad spectrum of agricultural crops, forests, and fruit trees, particularly during their larval stage when they affect the underground portions of plants [1]. Accurate species identification is crucial for agricultural researchers and scientists to develop effective control methods or management strategies [2,3,4,5,6]. Traditional insect classification methods, which rely on external features such as the head, thorax, abdomen, and propodites, present considerable challenges for non-specialists [7,8,9].

Fortunately, numerical taxonomy [10,11] has advanced significantly in recent decades. Today, it serves as a vital tool in modern systematics, especially in the study of fossil organisms within the Dicksoniaceae family [12]. With advancements in computer technology and image processing, geometric morphometrics—a statistical method for quantitatively describing organisms—has emerged as an effective and scientific approach in entomology, applicable to various insect species [13,14].

In Coleoptera taxonomy, interspecies differences or sexual dimorphisms are commonly studied using landmark analysis or morphological data such as hind wing shape [15,16]. At higher taxonomic levels, landmark data from the metendosternites of Scarabaeoidea beetles have been extracted and analyzed, demonstrating the utility of this structure for beetle systematists [17]. Through three-dimensional reconstructions, morphological variations in mandibles among three feeding types of beetles (omnivorous, phytophagous, and coprophagous) have been examined, leading to insights into the relationship between feeding habits and mandible structure [18]. Based on 19 landmarks of hind wings and 119 morphological characters from 81 beetle species, phylogenetic relationships have been investigated [15].

Numerous studies have predominantly focused on sister species or pairs of species. For instance, shape and size differences between polyphenic sister species within the genus *Onthophagus* Latreille, 1802 were studied using landmarks on the head, pronotum, and elytra [19]. However, these external traits alone were insufficient to distinguish between the two species unless primary sexual characteristics were considered [20]. In *Colophon* Gray, 1832 beetles, significant intersexual and interspecific variations in size and shape in the mandibles, heads, pronota, and elytra between two sympatric species (*Colophon haughtoni* Barnard, 1929 and *C. kawaii* Mizukami, 1997) showed significant sexual dimorphism. These variations allow for the accurate identification of males based on mandibles, heads, and pronota [21]. In two ground beetles (*Carabus auronitens* Fabricius, 1792 and *C. nemoralis* Müller, 1764), significant size differences were noted between sexes, with positive correlations observed between sexes and shapes [22]. Nikola et al. (2019) [23] found that allometric components significantly contributed to body shape differences between sexes, while taxonomic differences between two ground beetle species (*Carabus coriaceus cerisyi* Dejean, 1826 and *C. kollari praecellens* Palliardi, 1825) were influenced by additional factors and processes. For cantharid beetles (Cantharinae), hind wing shape differences among the genera appear more variable than within each genus, with geometric morphometrics revealing distinct variations for each species [24].

In the study of sexual dimorphism in insect species, scientists typically examine external morphological characteristics. For instance, in the case of the scarab beetle (*Adoretus sinicus* Burmeister, 1855), sexual differences were determined by the morphology of the terminal sternite and the length-to-width ratio of certain protarsomere segments [16]. The shape and size patterns of horns in *Ceratophyus rossii* Jekel, 1865 have been identified and modeled using both traditional and geometric morphometric methods [25]. Regarding ambrosia beetles (*Euwallacea fornicatus* Eichhoff, 1868 and *Euwallacea* sp. Bright, 2014), adult males of *E.* sp. exhibit significantly shorter body lengths, narrower head widths, and smaller pronotal widths compared to females. Additionally, the females of *E.* sp. have significantly narrower head widths and pronotal widths than female *E. fornicates*, while demonstrating significantly greater body lengths and ratios of body length to pronotal width [26].

In the field of ecology, the differences between insect ecotypes have been utilized to evaluate the relationship between morphological traits and ecological factors. For instance, morphological measurements were employed to compare intraspecific variations among dung beetles from different land use types, thereby elucidating the connection between morphological traits and land use patterns. The study demonstrated that intraspecific differences in dung beetle morphological traits vary across different land use types, and changes in land use can induce phenotypic plasticity [27]. By analyzing shape variation in 495 specimens from 38 species of dung beetles, it was found that taxonomic diversity is positively correlated with morphometric diversity [28]. Additionally, the shape variation and fluctuating asymmetry levels observed in the carabid beetle *Pterostichus melas melas* Creutzer, 1799 can serve as indicators of environmental stability [29]. In *Carabus aeruginosus* Fischer, 1822, certain environmental factors such as anthropogenic press and biotope vegetation significantly influenced body size variation, leading to sexual size and shape dimorphism [30]. Furthermore, latitudinal variation was evident in the sexual traits of 22 populations of the false blister beetle (*Oedemera sexualis* Marseul, 1877) [31]. However, the relationship between morphological diversity and species richness is not always straightforward; for example, studies on Lucanidae indicate that their morphological diversity does not necessarily correlate with species richness [32].

Species within the genus *Holotrichia* exhibit a distribution spanning the Palaearctic and Oriental regions. To date, over 500 species have been taxonomically recognized worldwide, among which 73 are documented in China [33]. The females and males of the three beetle species (*Holotrichia diomphalia* Bates, 1888, *H. titanis* Reitter, 1902, and *H. oblita* Faldermann, 1835) examined in this study exhibit no clear sexual dimorphism and possess a high degree of morphological similarity, making visual sex determination challenging. The hind wings of these beetles have traditionally not been considered a key diagnostic feature for species identification. Therefore, the objective of this study is to evaluate the utility of hind-wing morphology in distinguishing between these beetle species or their sexes through the extraction and analysis of landmarks on hind wings, which is a fast method that does not require taxonomic expertise.

## 2. Materials and Methods

### 2.1. Specimen Collection

The beetles utilized in this study were collected from the campus of Xinyang Agriculture and Forestry University in Henan Province, China. A total of 125 beetles (*H. diomphalia* male/female: 30/30, *H. titanis* male/female: 18/20, and *H. oblita* male/female: 17/10) were captured manually under the campus light lamps between 8:00 p.m. and 10:00 p.m. Specimens were immediately preserved in ethanol and subsequently transported to the laboratory. Taxonomic identification was conducted using established taxonomic keys, and sex determination was based on external morphological characters, including antennal size and external genitalia [34].

### 2.2. Slide Preparation

**Figure 1:**
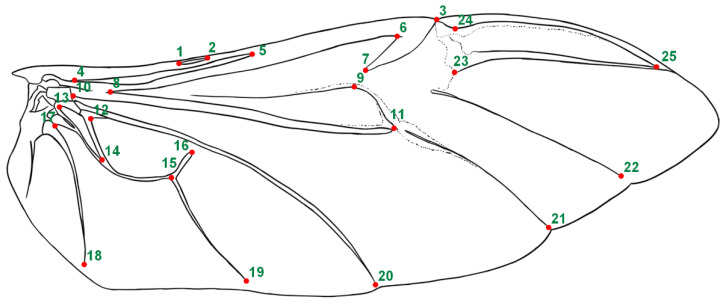
The right hind wing of beetles depicting 25 anatomical landmarks positioned at key morphological structures.

The right hind wing of each beetle was excised using a surgical-grade scalpel and precision forceps following forewing removal. The wing was immersed in boiling water for 3 min to achieve optimal flattening. Each prepared wing was mounted on a microscope slide and secured with a cover slide. High-resolution digital scanning (Epson Perfection v370 Photo, Seiko Epson Corporation (Suwa, Japan); resolution: 2400 dpi) was employed to capture wing morphology. The resulting images were processed to create standardized data files using TpsUtil v1.68 (Stony Brook, NY, USA), followed by digital landmark acquisition of 25 predefined anatomical points (shown in Figure 1) through TpsDig2 v2.31 [35]. Three independent digitizations were performed per wing to minimize measurement variability. Raw coordinate data were statistically averaged using Microsoft Excel v2007 (Redmond, WA, USA), generating consensus coordinates for subsequent morphometric analysis.

### 2.3. Geometric Morphometric Analysis

TPS files containing consensus coordinate data were imported into MorphoJ v1.06d for geometric morphometric processing [36]. Within the platform, all specimen coordinates underwent Procrustes superimposition to eliminate non-shape variation related to scaling, translational positioning, and rotational orientation. The resultant centroid size measurements and Procrustes-aligned coordinate set derived from these landmark data served as primary input variables for subsequent multivariate statistical analysis [37].

### 2.4. Size Variation

Wing size was quantified through centroid size measurements [36]. Interspecific variation in wing size was assessed through non-parametric analyses, initially employing the Kruskal–Wallis H-test, with post hoc pairwise evaluations conducted using Mann–Whitney U-tests (α = 0.05) in IBM SPSS Statistics v22.0 (IBM Corp., Armonk, NY, USA).

### 2.5. Shape Variation

Canonical variate analysis (CVA) was used to evaluate whether wing shape can discriminate between species and sexes, as well as to determine the most influential landmarks [37]. Shape variation was evaluated using permutation tests (10,000 rounds) based on Mahalanobis distances. Subsequently, a cross-validation test within discriminate function analysis (DFA) was conducted to assess classification accuracy. All analyses were performed using MorphoJ v1.06d software (Manchester, UK).

### 2.6. Sexual Shape Dimorphism

Differences in size between sexes for each species were assessed using the Mann–Whitney U-test in IBM SPSS Statistics v22.0. DFA was conducted to evaluate shape differences between sex samples based on Mahalanobis distances, using permutation tests with 10,000 iterations in MorphoJ v1.06d software. A cross-validation test was subsequently performed to assess the accuracy of classification [37].

### 2.7. Allometric Effects

To estimate allometric effects, the regression of Procrustes distance on centroid size was analyzed separately for each species and sex. Residuals from the regression of Procrustes coordinates on centroid size, derived from previous analyses, were utilized to assess shape differences independent of size effects. A cross-validation test was subsequently conducted to evaluate the accuracy of classification [37].

## 3. Results

### 3.1. Size Variation

**Figure 2:**
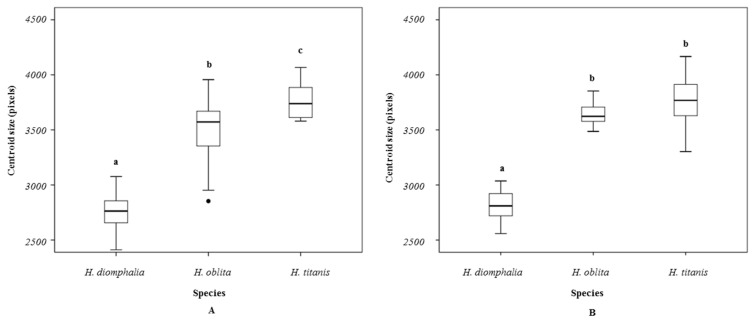
Boxplots showing centroid sizes of hind wings for each beetle species: (**A**) female, (**B**) male. Different letters indicate a statistically significant difference (Mann–Whitney *U*-test, *p* < 0.001).

**Figure 3:**
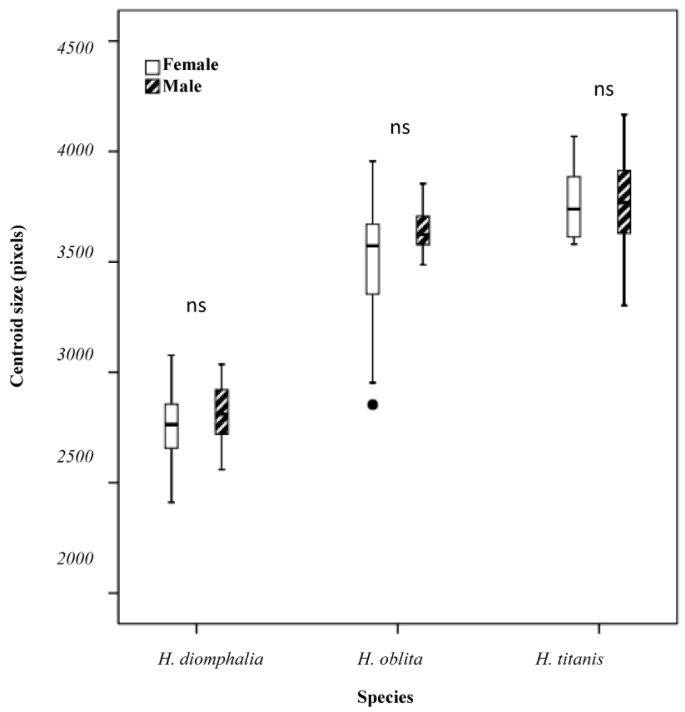
Boxplot showing centroid sizes of hind wings for each beetle species (ns: no significant differences).

Significant interspecific centroid size variation was detected in both female (Kruskal–Wallis H-test: χ^2^(2) = 48.384, *p* < 0.001) and male specimens (Kruskal–Wallis H-test: χ^2^(2) = 45.214, *p* < 0.001). Post hoc pairwise comparisons revealed significant sexual differences in centroid size across species, with the exception of male *H. oblita* and *H. titanis* (Mann–Whitney U-test, *p* = 0.055) (Figure 2). This interspecific variation was predominantly attributable to wing size dimorphism in female specimens. Notably, no significant sexual dimorphism in hind wing size was detected across the studied species (Mann–Whitney U-test, *p* > 0.05), indicating isometric hind wing development between sexes (Figure 3).

### 3.2. Shape Variation

Morphological variation manifested distinct distribution patterns along the first two canonical variate axes (CV1, CV2) in sex-specific analyses. Canonical variate analysis revealed distinct clustering patterns corresponding to species boundaries in both sexes.

In the female specimens, the two canonical variates CV1 = 74.69%, CV2 = 25.31% collectively explained 100% of morphological variance. Mahalanobis distances obtained by pairwise comparisons of all three species revealed significant differences (permutation test with 10,000 rounds in MorphoJ: *p* < 0.001), ranging from 10.02 (*H. oblita* and *H. diomphalia*) to 16.16 (*H. titanis* and *H. diomphalia*). Procrustes distances also showed significant differences between species (permutation test with 10,000 rounds in MorphoJ: *p* < 0.001) (Table 1).

**Figure 4:**
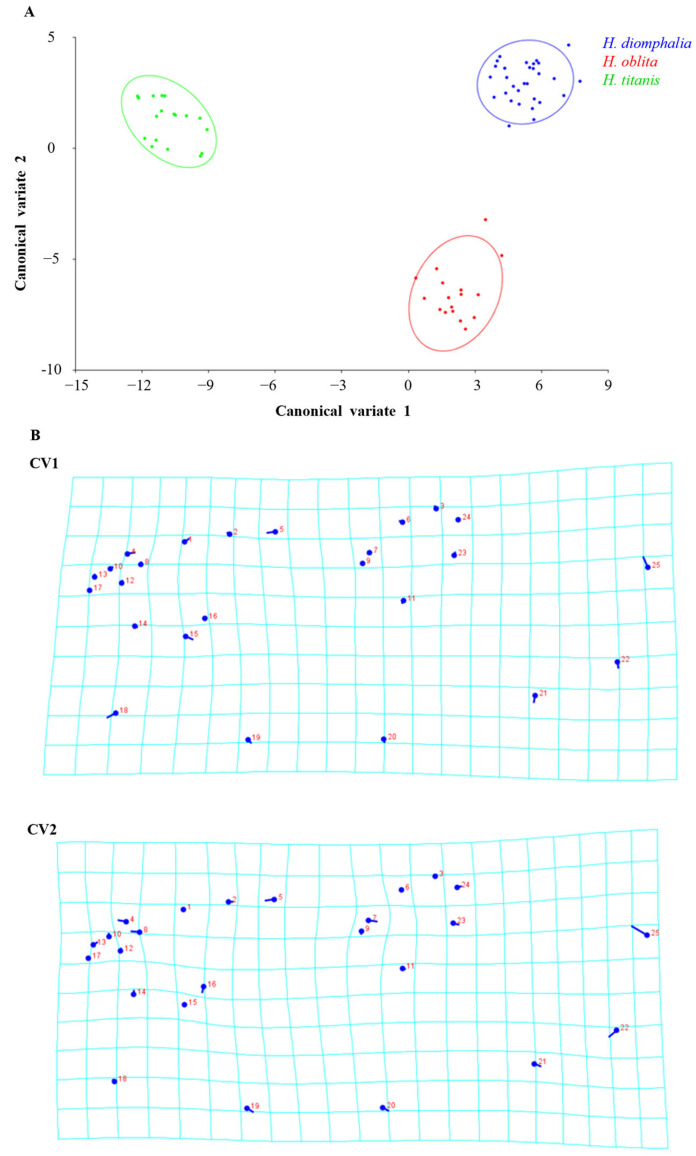
(**A**) Scatter plot showing the shape variation in hind wings from three female beetle species along the two canonical variate axes (CV1 and CV2) with 90% equal-frequency ellipses that are expected to contain approximately 90% of the data points. (**B**) Transformation grids illustrate the shape changes from overall means along the CV1 and CV2 axes in positive directions. Circles indicate the locations of the landmarks in the mean shapes of the samples; sticks indicate the changes in the relative positions of the landmarks.

Principal shape transformations along CV1 were primarily associated with landmarks 25, 18, 15, 4, 5, 21, 22, and 1, while CV2-related modifications involved landmarks 25, 22, 7, 8, 5, 4, 19, and 20 (Figure 4). The cross-validation test showed a percentage of corrected classification among species-classified specimens ranging from 52.94% to 88.89% by using a permutation test with 10,000 rounds in MorphoJ v1.06d (Table 2).

**Figure 5:**
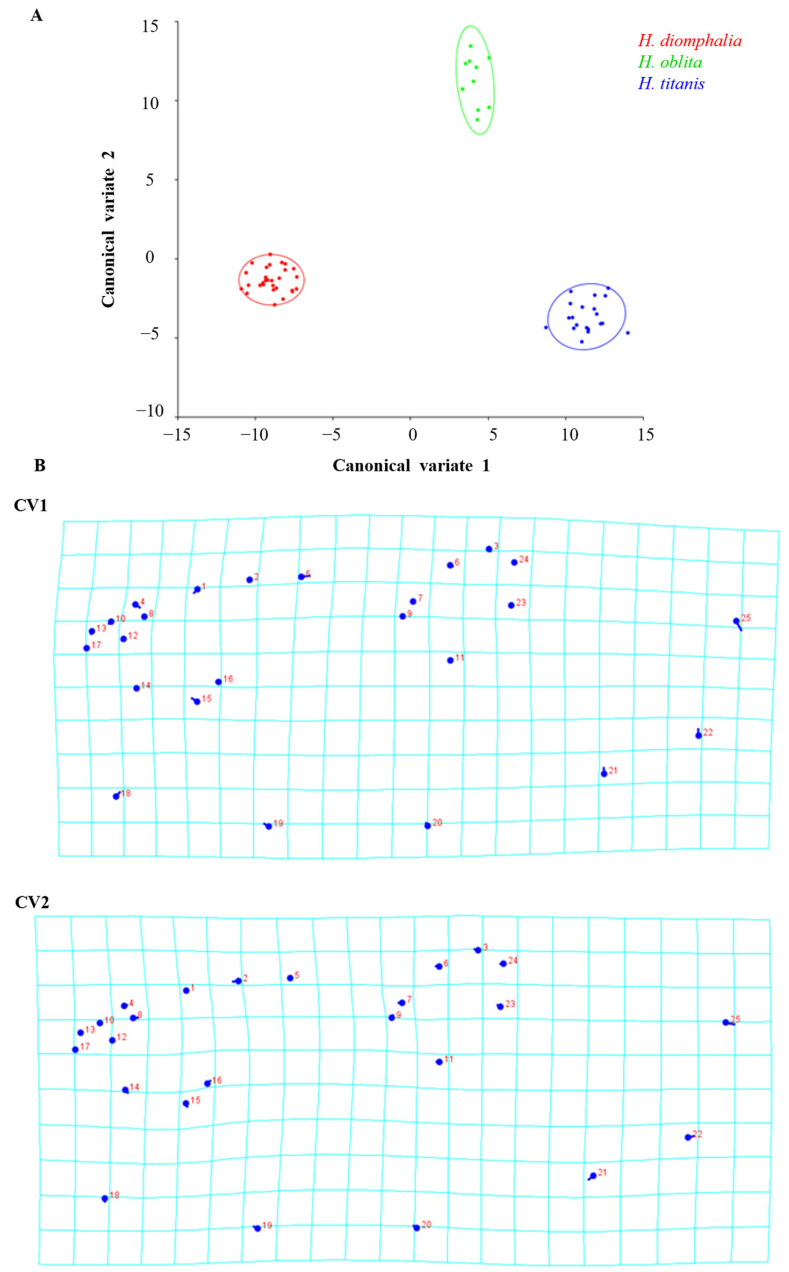
(**A**) Scatter plot showing the shape variation in hind wings from three male beetle species along the two canonical variate axes (CV1 and CV2) with 90% equal-frequency ellipses that are expected to contain approximately 90% of the data points. (**B**) Transformation grids illustrate the shape changes from overall means along the CV1 and CV2 axes in positive directions. Circles indicate the locations of the landmarks in the mean shapes of the samples; sticks indicate the changes in the relative positions of the landmarks.

In the male specimens, the two canonical variates CV1 = 76.49%, CV2 = 23.51% collectively explained 100% of the total morphological variation. Mahalanobis distances obtained by pairwise comparisons of all three species revealed significant differences (permutation test with 10,000 rounds in MorphoJ v1.06d: *p* < 0.001), ranging from 16.56 (*H. oblita* and *H. titanis*) to 20.46 (*H. titanis* and *H. diomphalia*). Procrustes distances also showed significant differences between species (permutation test with 10,000 rounds in MorphoJ v1.06d: *p* < 0.001), ranging from 0.03 (*H. titanis* and *H. oblita*) to 0.04 (*H. diomphalia* and *H. titanis*) (Table 3). Visualized shape changes along CV1 axis were found with landmarks 25, 5, 22, 15, 18, and 19, whereas shape changes along CV2 axis were 25, 22, 21, and 2 (Figure 5). The cross-validation test showed a percentage of classification/misclassification among species-classified specimens ranged from 60.00% to 86.67% by using a permutation test with 10,000 rounds in MorphoJ v1.06d (Table 4).

### 3.3. Sexual Shape Dimorphism

Mann–Whitney *U*-test showed no significant difference between females and males in one species (*H. diomphalia*: *p* = 0.179, *H. titanis*: *p* = 0.965, *H. oblita*: *p* = 0.264). The DFA in hind-wing shape between females and males revealed significant differences in *H. diomphalia* (*p* = 0.028), but no significance in *H. titanis* (*p* = 0.936) and *H. oblita* (*p* = 0.999). In this regard, the difference in females and males among one species could not be distinguished by hind-wing size or shape variation, although a higher percentage of correctly classified samples were identified by using a permutation test in MorphoJ v1.06d (Table 5).

### 3.4. Allometric Effects

The regression of the Procrustes coordinates according to centroid size among species showed a significant difference in female group (permutation test with 10,000 rounds in MorphoJ v1.06d: *p* < 0.001, allometry explained 16.92% of total shape variation) and male group (permutation test with 10,000 rounds in MorphoJ v1.06d: *p* < 0.001, allometry explained 25.35% of total shape variation). However, the relationship between shape and size within each species showed that there was no significant difference across all species (permutation test with 10,000 rounds in MorphoJ v1.06d: *p* > 0.05) (Table 6). Allometry causes no significant differences between sexes with intraspecies samples (Table 6).

The multivariate regression of the Procrustes coordinates according to the centroid size of hind wings within each species and between the sexes of each species performed using a permutation test with 10,000 rounds in MorphoJ v1.06d.

After removing the effect of size on shape variation, a cross-validation test showed a higher percentage of correctly classified specimens between species in female groups (94.12–100.00%), male groups (76.67–100.00%), and pooled sexes groups (71.05–89.47%) (Table 7). After removing the allometric effects, shape variation between sexes showed hind wing shape between females and males was not significantly different in two species, *H. titanis* and *H. oblita* (permutation 10,000 rounds in MorphoJ v1.06d: *p* > 0.05), except for *H. diomphalia* (*p* = 0.025) where the percentage of correctly classified samples for female and male specimens was 70.00% and 66.67% separately (Table 8).

**Figure 6:**
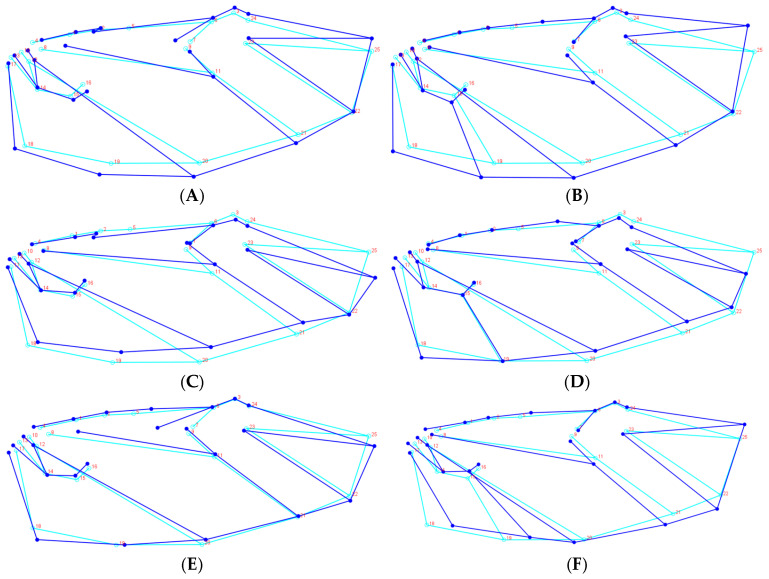
The variance accounted for by the first three principal components. (**A**) Female-PC1; (**B**) Male-PC1; (**C**) Female-PC2; (**D**) Male-PC2; (**E**) Female-PC3; (**F**) Male-PC3.

**Figure 7:**
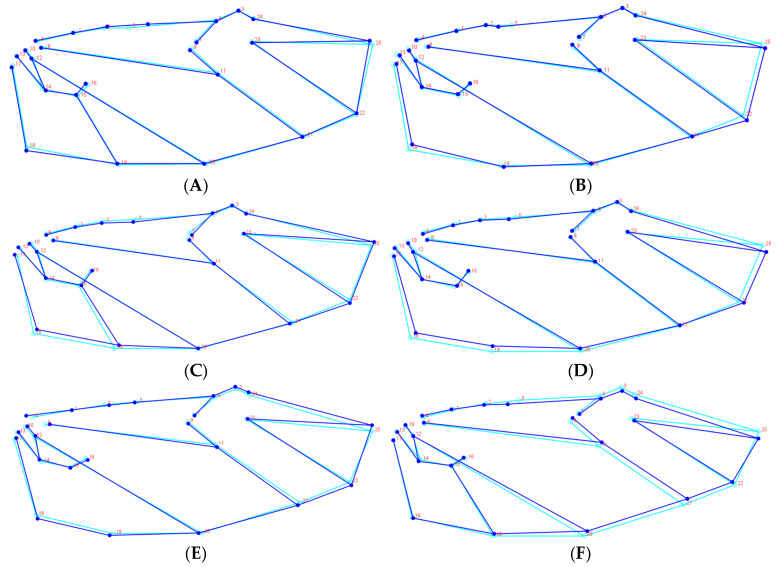
Wireframe diagrams showing the average position of landmarks and variation among specimens. (**A**) *H. diomphalia* female; (**B**) *H. diomphalia* male; (**C**) *H. oblita* female; (**D**) *H. oblita* male; (**E**) *H. titanis* female; (**F**) *H. titanis* male.

To remove the allometric component and size differences between these beetles, the results show that Principal Component Analysis (PCA) revealed multiple principal components for both male and female specimens. Among females, the first three principal components accounted for 21.62%, 17.48%, and 14.83% of the variance, respectively, while among males, these values were 26.03%, 12.73%, and 10.10% (Figure 6). The observed variations in wing apex angles, wing intersection points, and the position of the anal vein’s apical point influenced the contraction and expansion of the anal region as well as the elevation or depression of the wing apex. Overall, female wings exhibited a wider and shorter morphology compared to the more slender and elongated wings of males. In terms of interspecific differences, *H. oblita* females displayed narrow and elongated wings, whereas *H. diomphalia* females had a more rectangular wing shape. Among males, the degree of wing narrowness decreased in the order of *H. oblita*, *H. titanis*, and *H. diomphalia* (Figure 7).

## 4. Discussion

All scarab beetle specimens in this study were collected from the city of Xinyang (32°10′00″ N; 114°07′52″ E), Henan Province, China, a region characterized by a warm temperate humid monsoon climate. While these congeneric species are predominantly recognized as agricultural pests, certain larval stages (e.g., *Holotrichia diomophalia*) have shown emerging pharmacological interest as potential antifibrotic agents for hepatic disorders [38]. Compared to sexually dimorphic beetles such as *Colophon* spp. [21], *Holotrichia* species exhibit less pronounced sexual dimorphism. This investigation aimed to evaluate the diagnostic utility of hind wing morphology through geometric morphometric analysis for interspecific and sexual differentiation within the genus.

Morphometric landmarks were selected based on macroscopic visibility rather than phylogenetic significance [15,39]. This operational approach aligns with contemporary trends in automated insect identification while acknowledging that current automated image recognition systems remain agnostic to phylogenetic relationships. Analytical results revealed that landmarks demonstrating the greatest shape variation were predominantly located along the posterior wing margins across both sexes. Discriminant analysis achieved classification accuracy rates exceeding 94.12% in females and 76.67% in males following size correction; performance metrics were comparable to established morphological identification systems employing non-wing characteristics in Coleoptera [32].

Although hind wing parameters showed limited sexual discriminative power, the methodology establishes a supplementary diagnostic framework for *Holotrichia* species identification. This contributes to both traditional taxonomic practice and numerical classification systems, with potential applications in automated taxonomic identification systems. The findings contrast with documented sexual dimorphism patterns in other Coleoptera families where cephalic, pronotal, and elytral characteristics typically serve as primary diagnostic features [16,19,21,30,40,41,42].

## 5. Conclusions

The analysis demonstrated that hind-wing morphological variation provided sufficient discriminative power for taxonomic identification among the three congeneric species, while exhibiting limited efficacy in sexual differentiation. The results show that the female wings exhibited a wider and shorter morphology compared to the more slender and elongated wings of males. In terms of interspecific differences, *H. oblita* females displayed narrow and elongated wings, whereas *H. diomphalia* females had a more rectangular wing shape. Among males, the degree of wing narrowness decreased in the order of *H. oblita*, *H. titanis*, and *H. diomphalia*.

## Figures and Tables

**Table 1:** Differences in hind-wing shapes of female beetles among species with canonical variate analysis (CVA).

Species (Female)	*H. diomphalia*	*H. titanis*	*H. oblita*
*H. diomphalia*	-	0.04	0.04
*H. titanis*	**16.16**	-	0.04
*H. oblita*	**10.02**	**15.07**	-

Mahalanobis distances (bold) and Procrustes distances (normal). *p*-values of three species were statistically significant (permutation test with 10,000 rounds in MorphoJ v1.06d: *p* < 0.001).

**Table 2:** The percentage of classification/misclassification among species-classified female specimens by using a permutation test with 10,000 rounds in MorphoJ v1.06d.

Species (Female)	% of Classification/Misclassification Between Groups		
	*H. diomphalia*	*H. titanis*	*H. oblita*
*H. diomphalia*	**66.67% (20/30)**	33.33% (10/30)	
	**76.67% (23/30)**		23.33% (7/30)
*H. titanis*	22.22% (4/18)	**77.78% (14/18)**	
		**88.89% (16/18)**	11.11% (2/18)
*H. oblita*		35.29% (6/17)	**64.71% (11/17)**
	47.06% (8/17)		**52.94% (9/17)**

Corrected classification (bold) and misclassification (normal).

**Table 3:** Wing shape variations in male beetles among species with canonical variate analysis (CVA).

Species (Male)	*H. diomphalia*	*H. titanis*	*H. oblita*
*H. diomphalia*	-	0.04	0.04
*H. titanis*	**20.46**	-	0.03
*H. oblita*	**18.20**	**16.56**	-

Mahalanobis distances (bold) and Procrustes distances (normal). *p*-values of all three species were statistically significant (permutation test with 10,000 rounds in MorphoJ v1.06d: *p* < 0.001).

**Table 4:** The percentage of classification/misclassification among male species-classified specimens by using a permutation test with 10,000 rounds in MorphoJ v1.06d.

Species (Male)	% Classification/Misclassification Between Groups		
	*H. diomphalia*	*H. titanis*	*H. oblita*
*H. diomphalia*	**86.67% (26/30)**		13.33% (4/30)
	**60.00% (18/30)**	40.00% (12/30)	
*H. titanis*	30.00% (6/20)	**70.00% (14/20)**	
		**85.00% (17/20)**	15.00% (3/20)
*H. oblita*	20.00% (2/10)		**80.00% (8/10)**
		20.00% (2/10)	**80.00% (8/10)**

Corrected classification (bold) and misclassification (normal).

**Table 5:** The percentage of correctly identified samples between the females and males of each species identified by using a permutation test with 10,000 rounds in MorphoJ v1.06d.

Species	Female	Male
*H. diomphalia*	73.33% (22/30)	63.33% (19/30)
*H. titanis*	77.78% (14/18)	85.00% (17/20)
*H. oblita*	64.71% (11/17)	40.00% (4/10)

**Table 6:** Percentage of predicted size-related shape variation in hind wings in each beetle species and between sexes of each species.

Species	% of Predicted Within Species	*p*-Value	% Predicted Between Sexes	*p*-Value
*H. diomphalia*	2.89	0.084	3.02	0.068
*H. titaniis*	4.03	0.122	3.62	0.182
*H. oblita*	8.03	0.072	6.12	0.144

**Table 7:** The percentage of classification and misclassification of specimens between species identified using a permutation test with 10,000 rounds in MorphoJ v1.06d.

Groups	Species	% of Classification/Misclassification Between Species			*p*-Value (Parametric)
		*H. diomphalia*	*H. titanis*	*H. oblita*	
Females	*H. diomphalia*	**96.67% (29/30)**		3.33% (1/30)	0.320
		**100.00% (30/30)**	0.00% (0/30)		0.038
	*H. titanis*	5.56% (1/18)	**94.44% (17/18)**		0.038
			**94.44% (17/18)**	5.56% (1/18)	0.794
	*H. oblita*		5.88% (1/17)	**94.12% (16/17)**	0.794
		5.88% (1/17)		**94.12% (16/17)**	0.320
Males	*H. diomphalia*	**100.00% (30/30)**		0.00% (0/30)	0.403
		**76.67% (23/30)**	23.33% (7/30)		0.018
	*H. titanis*	10.00% (2/20)	**90.00% (18/20)**		0.018
			**80.00% (16/20)**	20.00% (4/20)	0.923
	*H. oblita*	10.00% (1/10)		**90.00% (9/10)**	0.403
			20.00% (2/10)	**80.00% (8/10)**	0.923
Pooled sexes	*H. diomphalia*	**85.00% (51/60)**		15.00% (9/60)	<0.001
		**73.33% (44/60)**	26.67% (16/60)		0.001
	*H. titanis*	28.95% (11/38)	**71.05% (27/38)**		<0.001
			**89.47% (34/38)**	10.53% (4/38)	<0.001
	*H. oblita*	18.52% (5/27)		**81.48% (22/27)**	<0.001
			18.52% (5/27)	**81.48% (22/27)**	<0.001

Corrected classification (bold) and misclassification (narrow).

**Table 8:** The percentage of correctly classified specimens between the sexes of each beetle species identified using a permutation test with 10,000 rounds in MorphoJ v1.06d.

Species	% of Correctly Classified Between Sexes (No. of Correctly Classified/Total No. of Specimens)		*p*-Value (Parametric)
	Female	Male	
*H. diomphalia*	70.00% (21/30)	66.67% (20/30)	0.025
*H. titanis*	38.89% (7/18)	45.00% (9/20)	0.934
*H. oblita*	41.18% (7/17)	30.00% (3/10)	0.983

## Data Availability

Data are unavailable.
